# Impact of Monetary Policy Uncertainty on R&D Investment Smoothing Behavior of Pharmaceutical Manufacturing Enterprises: Empirical Research Based on a Threshold Regression Model

**DOI:** 10.3390/ijerph182111560

**Published:** 2021-11-03

**Authors:** Jingyuan Yang, Ling Wang, Ziyuan Sun, Fangming Zhu, Yihui Guo, Yan Shen

**Affiliations:** School of Economics and Management, China University of Mining and Technology, Xuzhou 221116, China; yangjingyuan@cumt.edu.cn (J.Y.); cumtkjx218@126.com (L.W.); fming_zhu@163.com (F.Z.); guoyihui33@163.com (Y.G.); yan_shen98@163.com (Y.S.)

**Keywords:** R&D investment smoothing behavior, financing constraints, monetary policy uncertainty, pharmaceutical manufacturing enterprises, threshold regression model

## Abstract

R&D investment is the source of technological innovation of pharmaceutical enterprises, but it will be restricted by the funding level, especially in the context of major public health emergencies occurring more frequently, therefore exploring the impact of monetary policy uncertainty on the R&D investment smoothing behavior of pharmaceutical manufacturing enterprises has important theoretical and practical value. Based on the relevant data of Chinese pharmaceutical manufacturing enterprises from 2012 to 2018, this paper studies the impact of monetary policy uncertainty on R&D investment smoothing behavior of pharmaceutical enterprises, and investigates whether there is a threshold effect. First, our results demonstrate that the empirical test results of this article support the hypothesis of R&D investment smoothing behavior of pharmaceutical manufacturing enterprises. Second, there is a negative correlation between monetary policy uncertainty and R&D investment smoothing behavior, and the shorter the period is, the higher the financing constraints of pharmaceutical enterprises are, and the more obvious the negative correlation is. Third, financing constraints have a single threshold effect on the R&D investment smoothing behavior of pharmaceutical manufacturing enterprises, with a threshold of −13.7693. Moreover, this conclusion can better promote the virtuous circle of the real economy of financial and pharmaceutical manufacturing enterprises. It is recommended that pharmaceutical manufacturing enterprises establish and improve the enterprise R&D reserve system, reduce the risk of R&D investment, play the role of R&D smoothing, and realize the sustainable development of enterprise R&D.

## 1. Introduction

With the accelerating process of globalization and the evolution and development of society, major public health emergencies occur more frequently, which has a great impact on people’s lives and economic development, and it also has a strong stimulus to the development of the pharmaceutical industry. In the China–U.S. phase-one economic and trade agreement, the United States would strengthen the patent protection of drugs and expand the import of drugs and medical devices to China, which will have a profound impact on China’s biomedical industry. The development of the pharmaceutical manufacturing industry is facing more fierce international competition, the difficulty of drug R&D is increasing, and the transformation of medium and high-end medical devices is difficult. The COVID-19 epidemic that broke out in 2020 is a “black swan” encountered by the Chinese economy, which has undoubtedly brought a great impact on household consumption and enterprise production and investment. As a special industry directly related to the prevention and treatment of COVID-19, the strategic value of the pharmaceutical manufacturing industry is constantly improving with the development of the epidemic. Since the outbreak of COVID-19, many research institutions and pharmaceutical enterprises are accelerating the development of SARS-CoV-2 antibody drugs. However, the failure rate of new drug research and development in pharmaceutical manufacturing enterprises is very high. COVID-19 sorts out the time-consuming and uncertain factors of clinical approval in the pharmaceutical manufacturing industry, where R&D investment is ultimately determined by efficacy and clinical needs. The pharmaceutical manufacturing industry is a typical technology-driven industry with rapid growth in benefits, a broad market, and both economic and social benefits. Studying the R&D investment of pharmaceutical manufacturing enterprises is conducive to putting forward countermeasures and suggestions for the current technological innovation of the pharmaceutical manufacturing industry, further enhancing the independent innovation capabilities of Chinese pharmaceutical manufacturers, encouraging the research and development of original drugs, and enhancing the competitiveness of Chinese pharmaceuticals in the international market.

China has established a macro environment to encourage innovation and provide policy support for the innovation of pharmaceutical industry, such as the establishment of pharmaceutical parks to encourage innovation and various tax relief policies for innovative drug enterprises [1]. Under the loose monetary policy, China’s new drug investment market continues to be active. The monetary policy provides abundant fundraising channels for medicine, and maintains the continuous capital investment of innovative drugs, so that the investment in new drug R&D in China can produce a lot of returns. In recent years, the frequency of monetary policy regulation has increased significantly, and the issue of monetary policy uncertainty has become increasingly evident. Under the new global development trend, new methods and systems are needed to realize the healthy development of the industry [2,3]. The increase in monetary policy uncertainty has increased the difficulty for enterprises to predict the direction of policy, which in turn affects enterprise financial decision-making behavior [4]. The significant association between monetary policy and business investment has been confirmed [5]. Scholars have explored the specific path of enterprise investment under the influence of monetary policy from various angles, which include bank credit [6], commercial credit [7], labor costs, entrepreneur confidence [8,9], and investor sentiment. Most of these studies explore the impact of monetary policy tightening or easing on enterprise investment activities from a static perspective. However, the impact of uncertainty induced by monetary policy regulation on firms’ investment activities is relatively neglected. Besides, a few studies try to explore the relationship between monetary policy and innovative investment [10,11,12]. However, these studies are mainly devoted to answering how monetary policy tightening or easing affects R&D investment in the current period and does not involve the dynamic characteristics of enterprise R&D investment. For enterprises facing financing constraints, especially those enterprises that mainly rely on unstable resources for financing, relatively smooth R&D expenditure is an important financial policy to ensure the realization of the R&D investment strategy. Different from physical investment, the R&D investment of pharmaceutical manufacturing enterprises has stronger investment inertia and technology path dependence characteristics. The intertemporal dynamic characteristics of R&D investment have important implications for the effectiveness of firms’ innovation activities.

Based on this point, the paper focuses on solving the following three issues: do pharmaceutical manufacturing enterprises have R&D investment smoothing behavior? What are the distinctive characteristics of the impact of monetary policy uncertainty on enterprise R&D investment smoothing under different forecast periods? What is the impact of financing constraints on the weakening effect of monetary policy uncertainty on the smooth behavior of R&D investment?

Our research uses the empirical data of pharmaceutical enterprises to conduct an in-depth investigation of the impact of monetary policy uncertainty on pharmaceutical enterprises’ innovative investment smoothing behavior. There are four contributions to this paper. Firstly, based on 45 macro and financial variables in China for the period from January 2012 to December 2018, China’s monetary policy uncertainty is estimated using a high-dimensional factor model in a big data setting proposed by Jurado et al. [13]. Secondly, combining empirical testing and theoretical analysis, monetary policy uncertainty in enterprise into the R&D investment smoothing model to analyze the impact of monetary policy uncertainty on R&D investment smoothing of pharmaceutical enterprises, and further explores the differences in the impact under different forecast periods. Thirdly, in the further study of R&D investment smoothing under, the threshold effect of financing constraints is also discussed. Finally, relevant feasibility opinions based on the research results are put forward. Empirical research indicates that this research proves the hypothesis that pharmaceutical manufacturing enterprises have R&D investment smoothing behavior. Further, it is found that monetary policy uncertainty will weaken the R&D investment smoothing behavior of pharmaceutical manufacturing enterprises, and this weakening effect is stronger in high financing constraint enterprises.

This paper is organized as follows: Section 2 provides the background of monetary policy, mechanism analysis of its influence on firm innovation, and research hypotheses. Section 3 describes the methodology, variables, and model. Section 4 presents the monetary policy uncertainty measurement and baseline results. Section 5 presents the conclusions and policy implications. Section 6 presents the research limitations and outlook.

## 2. Theoretical Mechanism

Bloom et al. [14] recognized that the imperfections of the capital market will have an impact on R&D investment decisions, which will result in the dynamic volatility of R&D investment. Boeck and Feldkircher [15] found that market participants significantly underreact to a conventional monetary policy shock. In a study on the motivation of firms to increase their cash holdings to enterprise R&D investments, Gamba and Triantis [16] suggested that innovative firms hold cash out of a precautionary motive for uncertain future expenditures. Han and Qiu [17] assume that future cash flows cannot fully hedge against financial volatility risk, and the higher the volatility of cash flows, the larger the optimal precautionary cash holdings. That is, when R&D investment of enterprises is affected by cash flow fluctuations, sufficient cash holdings help the enterprise to maintain the stability of its R&D investment. Lyandres and Palazzo [18] tested the strategic motivation of innovative enterprises for cash holdings from both theoretical and empirical aspects. Because enterprises that frequently invest and innovate are more vulnerable and the potential for access to the capital market for external financing is weakened, innovative enterprises have higher cash holdings than other enterprises. At the same time, enterprises that hold large amounts of cash are more likely to invest in R&D, and this can also strengthen the ability of enterprises to deal with the uncertain monetary policy [19].

In studies related to firms smoothing R&D investments through cash holdings, Almeida et al. [20] and Brown and Petersen [21] have shown that firms generally use cash holdings to smooth R&D investments and that reduced cash holdings make R&D investments more volatile. Taewon Kang et al. [22] found that it is the heterogeneity of the technological capabilities that leads to the contradictory conclusions in the R&D investment literature. It was also found that the cash flow effect was magnified due to technical capabilities. When there is a positive impact, as sales increase, technological capabilities would amplify the volatility of R&D investment. When there is a negative impact, technical capabilities would offset the volatility of R&D investment and exhibits persistence. Coldbeck and Ozkan [23] used a partial adjustment model to compare research and development investment and capital investment behavior of American enterprises from 2002 to 2016. It was found that no matter whether in capital investment or R&D investment, there are adjustment behaviors to target investment (optimal level). Brown and Petersen [21] proposed that high adjustment costs are the main driving force for R&D investment smoothing behavior. The research of Kim et al. [24] and Almeida et al. [20] found that financing-constrained enterprises may not use cash holdings to completely smooth R&D investment. The reason is that on one hand, large current cash holdings imply a reduction in current investments; on the other hand, due to the existence of agency costs, enterprise cash holdings generate higher interest taxes than personal income taxes. At the same time, if the current cash holdings are exhausted, it also means that the cash available for smooth R&D in the future will decrease. In the work of Brown et al. [25,26] because of the complex impact of financing constraints on R&D investment of non-US enterprises, they further studied the R&D situation of European enterprises based on a large sample. They found that the availability of funds has a direct impact on R&D activities in the case of controlling the total cash “reserves” for R&D investment and external equity financing. Enterprises will use internal cash flow and external funds to “smooth” R&D investment. At the same time, external equity financing has an increasingly important influence on R&D investment activities.

In summary, enterprises have sufficient incentives to smooth R&D investment through cash holdings. However, due to the high cost of cash holdings, there is still disagreement whether they have a significant smoothing effect on R&D investment. Therefore, hypothesis H1 is as follows:

**Hypothesis** **1** **(H1).** *Pharmaceutical manufacturing enterprises have R&D investment smoothing behavior.*

Stable expectations of policy have an important impact on firms’ investment decisions, and uncertainty will make firms’ investment decisions cautious [27]. Changes in monetary policy affect the investment opportunities and financing constraints of enterprises through multiple channels such as capital costs and asset prices, which eventually lead to adjustments in firms’ investment decisions [28,29,30]. Monetary policy is an important tool for the government to regulate macroeconomic operations. Specifically, monetary policy uncertainty affects firms’ R&D investment smoothing decisions in the following ways.

First of all, the uncertainty of monetary policy increases the risk expectations of enterprise capital interruption and strengthens the motivation of enterprise R&D investment smoothing behavior. Studies have shown that uncertain monetary policy regulation makes it difficult for financial institutions to form expectations of stable policy direction, which increases the difficulty of credit maturity management and capital management for financial institutions [31]. To avoid risks, financial institutions will reduce credit supply and shorten the credit period [32]. For long-term innovation activities, monetary policy uncertainty caused banks to shorten the credit period [33]. It makes it difficult for enterprises to obtain stable support from external funding during the R&D cycle, thus making them more dependent on the use of liquidity reserves to provide complimentary financial support for R&D investments. Besides, Kulatila-ka and Perotti [34], Weeds [35] emphasized the importance of growth options in R&D investment. That is, the earlier the R&D investment is made, the more rewarding the technological innovation results will be to the R&D firm.

Second, monetary policy uncertainty increases the difficulty for firms to grasp R&D investment opportunities and reduces their willingness to innovate in R&D [36], which in turn weakens the motivation for R&D investment smoothing behavior. The cost of funds is an important channel for monetary policy to affect enterprise investment [37]. Easing or tightening of monetary policy affects the cost of capital of enterprises, which in turn changes the net present value of investment projects calculated based on the cost of capital, and ultimately affects the judgment of enterprises on investment opportunities. Generally speaking, the impact of capital cost changes on the net present value of investment is positively correlated with the project investment cycle. That is, the net present value of long-term investment projects is more sensitive to capital cost changes. Frequent adjustments in monetary policy make it difficult for enterprises to judge investment opportunities based on the net present value of investments. This impact will be more obvious in innovative activities with long cycles and high uncertainties. This reduces the willingness of enterprises to innovate in R&D and inhibits the smooth progress of R&D investment.

Finally, monetary policy uncertainty affects firms’ R&D investment smoothing behavior by affecting managers’ sentiment, and the sentiment of economic agents plays an important role in the process of influencing enterprise innovation investment [38]. Behavioral finance theory believes that uncertainty tends to shape management’s pessimistic expectations, and management is worried about future financial difficulties. This sentimental effect will make management decision-making tend to be conservative and reduce investment, especially R&D investment with high-risk characteristics [39]. At the same time, rising risk aversion will strengthen the preventive motivation of enterprises to hold liquid assets and weaken the willingness of enterprises to use existing liquid assets to provide supplementary support for R&D investment. Therefore, hypothesis H2 is as follows:

**Hypothesis** **2** **(H2).** *Monetary policy uncertainty will weaken the R&D investment smoothing behavior of pharmaceutical manufacturing enterprises.*

Real option theory holds that when the uncertainty of economic policy increases, enterprises need to make trade-off choices about innovation behavior. The choice of enterprises is influenced by many factors, one of which is financing constraints. On one hand, there is no mandatory requirement for enterprises to report their innovation status in China. For the purpose of trade secret protection, many enterprises do not take the initiative to report information about innovation activities. This behavior intensifies the information asymmetry between creditors, investors, and enterprises [40], and increases the external financing cost of enterprises. On the other hand, innovation activities need continuous investment. If enterprises use external financing, it need to pay a lot of interest and dividend costs [41]. In the period of monetary policy uncertainty, it is more difficult for enterprises with strong financing constraints to obtain funds from outside. Enterprises are more inclined to hold cash and financial assets with the motivation of Preventive Savings, and reduce R&D investment. Enterprises with stronger financing constraints are more sensitive to monetary policy uncertainty [42]. Therefore, financing constraints will become an obstacle to the effect of monetary policy uncertainty on enterprise R&D investment. Many research results show that financing constraints are the most fundamental reason for determining the internal cash holdings of enterprises [17]. To buffer the R&D cash flow influenced by financing constraints, enterprises facing financing constraints are more inclined to strengthen the management of assets liquidity. Therefore, hypothesis H3 is as follows:

**Hypothesis** **3** **(H3).** *Compared with low financing constraints, the weakening effect of monetary policy uncertainty on R&D investment smoothing behavior of high financing constraints enterprises is stronger.*

**Figure 1:**
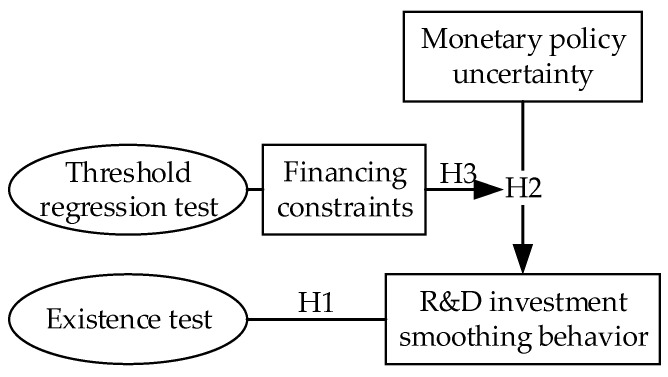
The theoretical model of the paper.

The theoretical model of this paper is shown in Figure 1.

## 3. Research Design and Main Model

### 3.1. Selection of Samples

Our research uses publicly listed pharmaceutical manufacturing enterprises in China’s Shanghai and Shenzhen A-share markets from 2012 to 2018 as research samples. Since the R&D investment in the model needs to take the previous year’s data, the main observation interval is 2013–2018. Firstly, there are two reasons for setting the window period to 2012–2018: (1) the window period selected can identify policy effects better and exclude the noise interference that may exist for a long period; (2) the data of R&D investment before 2012 are missing too much information. Secondly, our research refers to the industry classification standard of listed enterprises in the China Securities Regulatory Commission and selects pharmaceutical manufacturing enterprises in the manufacturing industry. We deal with data in the following steps, excluding (1) listed firms after 2012, (2) sample firms of ST or *ST on the A-share market, and (3) firms with missing data. Finally, due to the lack of R&D investment data of some enterprises, the balanced panel data with 126 pharmaceutical manufacturing enterprises were obtained.

The data sources of this paper are as follows. Firm R&D investment intensity, cash holdings, and other firm-level characteristic variable data are from the CSMAR database (http://cn.gtadata.com, access date: 15 October 2020). The CSMAR (China Stock Market & Accounting Research Database) database is a research accurate database in the economic and financial field which is developed based on China’s actual national conditions. The data used for the monetary policy uncertainty measures are obtained from the CEIC database (https://cas.ceicdata.com, access date: 15 October 2020) and the CElnet statistical database (https://db.cei.cn, access date: 15 October 2020).

### 3.2. Definition of Monetary Policy Uncertainty

Referring to the method of Jurado et al. [13] to measure economic uncertainty, our article uses China’s monetary policy and macroeconomic variables to measure China’s monetary policy uncertainty. The specific uncertainty theoretical model is as follows:

The uncertainty $U_{jt}^{y}(h)$ of the variable $y_{jt}\in Y_{t}$ in the future h period can be expressed as the degree of conditional deviation between the expected value $E[y_{jt+h}|I_{t}]$ predicted based on the t period information $I_{t}$ and the true value $y_{jt+h}$ in the future h period. It can be expressed as:

(1) $$U_{jt}^{y}(h)=\sqrt{E[{\left(y_{jt+h}-E[y_{jt+h}|I_{t}]\right)}^{2}|I_{t}]}$$

Among them, $E[\cdot |I_{t}]$ represents the expected value of the condition based on the information $I_{t}$ of the t period. If all variables in the set $Y_{t}$ of total variables related to monetary policy are added up with a certain weight $w_{t}$, the uncertainty of monetary policy can be obtained. It can be expressed as:

(2) $$U_{t}^{y}(h)=p\lim_{N_{y}\to \infty }\sum_{j=1}^{N_{y}}w_{j}U_{jt}^{y}(h)\equiv E_{w}[U_{jt}^{y}(h)]$$

**Figure 2:**
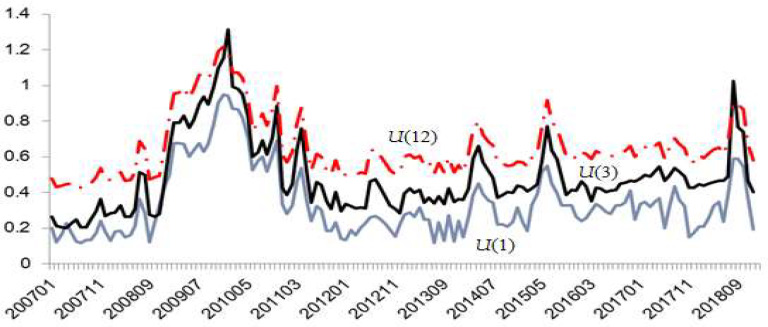
Explanation of variables used in constructing China’s monetary policy uncertainty index.

By extracting common factors from all conditional volatility by weighted average, the uncertainty index of monetary policy is obtained. There are 14 variables related to monetary policy, and 31 other macroscopic variables, for a total of 45 variables. The specific situation is shown in Table A1. All data used are monthly data. The time interval is 200701–201812. All data come from the CEIC database (https://cas.ceicdata.com, access date: 15 October 2020) and the CElnet statistical database (https://db.cei.cn, access date: 15 October 2020). Referring to Jurado et al. [13], this paper obtains China’s monetary policy uncertainty index with forecast periods of 1 month ($U_{1}$), 3 months ($U_{3}$), and 12 months ($U_{12}$), as shown in Figure 2. It can be seen from Figure 2 that the uncertainty indexes with forecast periods of 1 month, 3 months, and 12 months increase sequentially. This is because the longer the forecast time, the greater the accumulated uncertainty.

### 3.3. Other Variables’ Definitions

Table 1 shows additional variables. The dependent variable is R&D investment (RD). This paper mainly uses the R&D investment as a proxy variable for the R&D input of China’s publicly listed pharmaceutical manufacturing firms. Changes in cash holdings (${\;}_{△}Cash$) are measured using the ratio of changes in cash and cash equivalents to total assets for the period. The coefficient of the interaction term ($U\times_{△}Cash$) reflects the moderating effect of monetary policy uncertainty on the smoothing behavior of pharmaceutical enterprises’ innovative investment. At the same time, financing constraints (ICR) are interest guarantee multiples. Existing literature has studied the influencing factors of firm innovative investment [43,44,45]. The remaining control variables mainly include asset liability ratio, return on total assets, enterprise growth, proportion of the largest shareholder, and enterprise size.

### 3.4. Empirical Model

Bond and Meghir [46] proposed to use dynamic investment models to study the impact of enterprise internal cash flow on investment. In recent years, scholars have continuously improved the model. Brown and Petersen (2011) [21] discuss and estimate a dynamic R&D model with financial variables that is based on the Euler equation developed by Bond and Meghir (1994) [46] to study fixed investment under the assumption of quadratic adjustment costs. We estimate a similar dynamic innovative investment specification, but we take into account changes in cash holdings to directly explore the use of cash reserves for innovative investment smoothing. The specification is:

(3) $$\begin{array}{ll} {RD}_{i,t\;}= & \alpha_{1}+\beta_{1}{RD}_{i,t\;-\;1}+\beta_{2}{RD}_{i,t\;}^{2}+{\beta_{3}}_{△}Cash_{i,t\;}+\beta_{4}{LEV}_{i,t}+\beta_{5}{ROA}_{i,t}\\ & +\beta_{6}{Growth}_{i,t}+\beta_{7}{TOP1}_{i,t\;}+\beta_{8}{Size}_{i,t}+\varepsilon \end{array}$$

In order to further study the impact of monetary policy uncertainty on the smoothing behavior of R&D investment of pharmaceutical manufacturers, the variables of monetary policy uncertainty were added to Equation (3) to test, and the following model was established:

(4) $$\begin{array}{ll} {RD}_{i,t\;}= & \alpha_{i}+\beta_{1}{RD}_{i,t\;-\;1}+\beta_{2}{RD}_{i,t\;}^{2}+{\beta_{3}}_{△}Cash_{i,t\;}+\beta_{4}U_{i,t}\times_{△}Cash_{i,t}+\beta_{5}U_{i,t}\;\\ & +\beta_{6}{LEV}_{i,t}+\beta_{7}{ROA}_{i,t}+\beta_{8}{Growth}_{i,t}+\beta_{9}{TOP1}_{i,t\;}+\beta_{10}{Size}_{i,t}+\varepsilon \end{array}$$

This paper adopts the method of Hansen [47] and Yeh [48] to construct Equation (5). Equation (5) is constructed in the paper to test hypothesis H2. Specifically, a threshold regression model is assumed to exist as follow. Among them, the dependent variable is enterprise R&D investment (RD), the threshold variable is financing constraints (ICR), I is indicative function, T is the threshold to be estimated, α is the intercept term estimated by the model, $\beta_{i}$ is the regression coefficient of each variable, and ε is the random interference term. The remaining variables are the control variables.

(5) $$\begin{array}{ll} {RD}_{i,t\;}= & \alpha_{1}+\beta_{1}{RD}_{i,t\;-\;1}+\beta_{2}{RD}_{i,t\;}^{2}+{\beta_{3}}_{△}Cash_{i,t\;}I(ICR\leq T)+{\beta_{4}}_{△}Cash_{i,t\;}I(ICR\text{>}T)\\ & +\beta_{5}{LEV}_{i,t}+\beta_{6}{ROA}_{i,t}+\beta_{7}{Growth}_{i,t}+\beta_{8}{TOP1}_{i,t\;}+\beta_{9}{Size}_{i,t}+\varepsilon \end{array}$$

## 4. Empirical Analysis

### 4.1. Descriptive Statistics

Table 2 shows the descriptive statistical information of all variables, and the results are presented below. Firstly, the minimum value of RD is 0.000 and the maximum value is 11.940. This shows that the R&D investment intensity of pharmaceutical manufacturing enterprises is highly variable. Secondly, the mean value of RD (2.146) is greater than the median (1.895), indicating that half of the pharmaceutical manufacturing enterprises have lower R&D investment intensity than the average. Then, the maximum value of ${\;}_{△}Cash$ is 0.4397, the minimum value is −0.3991, and the standard deviation is 0.0942. This shows that the cash adjustment behavior among the sample enterprises has strong individual characteristics. Finally, in terms of monetary policy uncertainty, the average $U_{1}$ value of 0.240 is significantly smaller than $U_{3}$, and the average $U_{3}$ value of 0.417 is significantly smaller than $U_{12}$. This shows that the longer the forecast period of monetary policy uncertainty, the greater the volatility and sensitivity of monetary policy.

### 4.2. Research on R&D Investment Smoothing Behavior

Table 3 presents the full sample estimation results, where Equation (3) shows the estimation results without monetary policy uncertainty. It shows that due to the strong durability of R&D activities, the regression coefficient of lagging ${RD}_{t-1}$ is close to one. The coefficient of ${\;}_{△}Cash$ in columns A is −1.151, and it is significant at the level of 1%, indicating that changes in cash holdings (${\;}_{△}Cash$) have a significant negative effect on the R&D investment of pharmaceutical manufacturing enterprises. Enterprises have R&D investment smoothing behavior, which supports H1.

### 4.3. Research on the Impact of China’s Monetary Policy Uncertainty on Chinese Pharmaceutical Manufacturing Enterprises’ R&D Investment Smoothing Behavior

Table 4 presents the full sample estimation results; it is found that the $U_{1}\times_{△}Cash$ and $U_{3}{\;}_{△}Cash$ estimated coefficients of the cross terms of monetary policy uncertainty and changes in cash holdings are both significantly positive, which is opposite to the estimated coefficients of ${\;}_{△}Cash$. These three estimated coefficients are all significant at the level of 1%. It indicates that monetary policy uncertainty (with a forecast period of 1 and 3 months) weaken the R&D investment smoothing behavior of firms. However, the $U_{12}\times_{△}Cash$ estimated coefficients of the cross terms of monetary policy uncertainty and changes in cash holdings are insignificant. This indicates that monetary policy uncertainty with a forecast period of 12 months does not affect firms’ R&D investment smoothing behavior.

As described in the theoretical analysis, monetary policy uncertainty reinforces firms’ risk aversion needs and makes it more difficult for firms to grasp R&D investment opportunities, thus creating a disincentive for R&D investment smoothing behavior. From the estimation results, we can find that the weakening effect of monetary policy uncertainty on firms’ R&D investment smoothing behavior gradually decreases with the extension of the forecast period. A possible reason is that the accuracy of monetary policy forecasts will decrease over time when enterprises predict that there will be long-term monetary policy uncertainty; they cannot predict the direction of monetary policy changes. For pharmaceutical manufacturing enterprises, R&D investment is crucial to the development of the enterprise, and R&D investment has the particularity that it cannot be interrupted. Therefore, pharmaceutical manufacturing enterprises need to maintain R&D investment. Therefore, when there is long-term uncertainty in forecasting monetary policy, the weakening effect of monetary policy uncertainty on the R&D investment smoothing behavior will decrease.

### 4.4. Threshold Regression Model Test

The estimated value of each threshold is tested as follows: first, testing the threshold effect. Second, determining the number of observation thresholds under the threshold effect. Finally, the threshold value in the threshold regression model is solved with the sequential estimation method proposed by Hansen [47]. On this basis, the confidence interval of the threshold is constructed.

From the results in Table 5, at the 5% significance level, a single threshold passed the test and a double threshold failed. The estimated value of the single threshold is −13.7693, which is within the 95% confidence interval (−40.0616, −12.6846), and the double threshold effect is not significant, so there is no need to test the truth of the threshold estimate.

As is shown in Table 6, the 95% confidence interval of the threshold estimate is the interval formed by the value of the critical value when the LR value has less than a 10% significance level. The estimated threshold value is equal to the true value, whose threshold value is true and effective. According to the conclusion of the threshold regression model, for the impact of financing constraints on the R&D investment smoothing behavior of Chinese pharmaceutical manufacturing enterprises there exists a threshold effect.

RDTable 7 is the empirical results of Equation (5). The smaller the ICR, the bigger the financing constraints. It can be seen from Table 7 that the absolute value of the regression coefficient of ICR≤−13.7693 is greater than that of ICR >−13.7693. In addition, the R&D investment and changes in cash holdings are both significant at the 5% level. This shows that the greater the financing constraints faced by enterprises, the stronger the motivation for R&D investment smoothing behavior; hypothesis H2 passes the verification.

From the column Full sample in Table 8, from the column ICR≤−13.7693 and ICR >−13.7693 in Table 8, the estimated coefficients of ${\;}_{△}Cash$ and U in ICR≤−13.7693 are both greater than ICR >−13.7693. The economic implication is that, compared with low financing constraints, monetary policy uncertainty has a relatively larger inhibitory effect on the R&D investment smoothing behavior of enterprises facing high financing constraints.

Compared with enterprises with low financing constraints, the uncertainty of monetary policy has a stronger impact on the smoothing behavior of R&D investment of enterprises with high financing constraints. This indicates that financing constraints are the key factor restricting enterprises’ R&D investment, which is mutually confirmed with the research conclusion of Lee and Choi [49]. Enterprises facing severe financing constraints have difficulty in obtaining funds from outside and have to pay a higher premium. In a fiercely competitive environment, pharmaceutical manufacturing enterprises need to invest in innovation to gain a competitive advantage. Once the innovation activity is interrupted, the enterprise will not only face the risk of being robbed of market share, but may even be forced to delist. Therefore, in the fierce product market competition environment, compared with enterprises with low financing constraints, enterprises with high financing constraints have stronger incentives to use cash to smooth innovative investment. Innovation in pharmaceutical manufacturing enterprises has a high degree of uncertainty. The stronger the financing constraints faced by pharmaceutical manufacturing enterprises, and the more frequent the changes in monetary policy, the greater the likelihood that they will suffer from innovation failure. As monetary policy uncertainty increases, firms are more reluctant to invest in R&D [50]. Therefore, in enterprises with high financing constraints, the monetary policy uncertainty has a stronger weakening effect on the R&D investment smoothing behavior of pharmaceutical manufacturing enterprises.

## 5. Conclusions and Policy Implications

Because of the high innovation adjustment cost and the unstable financing, it is of great significance for pharmaceutical manufacturing enterprises to keep relatively stable R&D expenditure by smoothing R&D when they deal with short-term financial crises or financial turbulence. Based on the 2012–2018 A-share listed pharmaceutical manufacturing enterprises in China as a sample, after verifying the smooth behavior of Pharmaceutical Manufacturing Enterprises’ R&D investment, this paper investigates the effect of financing constraints on R&D investment smoothing behavior and monetary policy uncertainty with the moderating effect of R&D investment smoothing behavior. Conclusions are as follows: (1) pharmaceutical manufacturing enterprises have R&D investment smoothing behavior; (2) monetary policy uncertainty has a negative moderating effect on the impact of R&D investment smoothing behavior. Meanwhile, it has different effects on the R&D investment of the pharmaceutical enterprises in different forecast periods. Specifically, short-term monetary policy uncertainty has the strongest weakening effect on the R&D investment smoothing behavior; (3) financing constraints have a threshold effect on the R&D investment smoothing behavior of pharmaceutical manufacturing enterprises. Compared with low financing constraints, pharmaceutical manufacturing enterprises with high financing constraints have stronger R&D investment smoothing behavior, and the monetary policy uncertainty has a stronger weakening effect on the R&D investment smoothing behavior. Studies in the paper both enrich the research literature on the micro effects of monetary policy and provide useful references for the search for factors influencing R&D investment smoothing behavior of pharmaceutical manufacturing enterprise at the macro level.

There are also some valuable managerial implications for government and pharmaceutical manufacturing enterprises. In the face of rising economic uncertainty at home and abroad, the strengthening of monetary policy uncertainty is unavoidable. The Chinese government should strengthen the transparency of monetary policy regulation and reduce the negative impact of monetary policy uncertainty. At the same time, the People’s Bank of China should strengthen the necessary communication with the public about policy information and guide the public’s forward-looking expectations about the way monetary policy is regulated and the ideas of regulation. For Chinese pharmaceutical manufacturing enterprises, first of all, they should pay attention to the impact of adjustment cost on the effectiveness of R&D activities and strengthen the dynamic management of R&D investment. Secondly, it is recommended to establish and improve the enterprise R&D reserve system to support the sustainability of the R&D of pharmaceutical manufacturing enterprises. Finally, under financing constraints, pharmaceutical manufacturing enterprises should strengthen current asset management and improve cash flow conditions. Pharmaceutical manufacturing enterprises should strive to develop multiple financing channels, and actively raise funds for R&D by introducing venture capital or realizing the combination of early R&D and sales.

## 6. Research Limitations and Outlook

This study has several limitations and future research directions. First, due to the complexity of the model and the difficulty of obtaining data, this study does not further strengthen the analysis of the impact of monetary policy uncertainty on the smoothing of R&D investment of pharmaceutical enterprises. In the following research, we will consider more influencing factors and conduct empirical analysis. Second, this article mainly studies the impact of monetary policy uncertainty on the entire pharmaceutical industry, and then ignores the classification of companies with different characteristics. In the follow-up research, we will classify companies and conduct a more detailed analysis which can make relevant recommendations with more targeted and practical significance.

## Figures and Tables

**Table 1:** Definition and description of main variables.

Variables	Description
RD	Innovative investment: R&D investment to sales revenue
${\;}_{△}Cash$	Changes in cash holdings: Changes in cash and cash equivalents/Total assets
ICR	Financing constraints: interest guarantee multiples
LEV	Asset liability ratio: The ratio of total liabilities to total assets
ROA	Return on total assets: Net profit/total assets
Growth	Enterprise growth: Operating income growth rate
TOP1	Proportion of the largest shareholder
Size	Enterprise size: Natural logarithm of the enterprise’s total assets at the end of the period

**Table 2:** Descriptive statistics.

Variables	Observations	Standard Deviation	Mean	Maximum	Minimum	Median
RD	774	1.491	2.146	11.940	0.000	1.895
${\;}_{△}Cash$	774	0.094	0.007	0.440	−0.399	0.006
ICR	774	502.609	28.944	11,623.759	−832.623	3.241
$U_{1}$	774	0.046	0.240	0.315	0.173	0.246
$U_{3}$	774	0.030	0.417	0.461	0.363	0.417
$U_{12}$	774	0.015	0.578	0.602	0.552	0.577
LEV	774	0.192	0.323	0.968	0.015	0.301
ROA	774	0.067	0.065	0.340	−0.800	0.061
Growth	774	0.635	0.214	14.295	−0.645	0.146
TOP1	774	13.936	33.821	89.090	6.800	31.940
Size	774	0.404	9.316	10.634	8.180	9.302

**Table 3:** The results of R&D investment smoothing behavior of pharmaceutical manufacturing enterprises.

Variables	Equation (3)
	RD
${RD}_{t-1}$	0.764 ***
	(3.99)
${RD}_{t}^{2}$	−0.046
	(−1.29)
${\;}_{△}Cash$	−1.151 ***
	(−4.59)
LEV	−0.284
	(−1.23)
ROA	−0.096
	(−0.17)
Growth	0.157 ***
	(3.80)
TOP1	−0.002
	(−0.33)
Size	0.126
	(0.56)
Constant	−0.158
	(−0.07)
observations	774
${Adj\;R}^{2}$	0.185

Note: *** indicate significance levels of 1%.

**Table 4:** The impact of monetary policy uncertainty on the R&D investment smoothing behavior.

Variables	Equation (4)		
	RD		
${RD}_{t-1}$	0.700 ***	0.738 ***	0.756 ***
	(3.87)	(3.88)	(3.96)
${RD}_{t}^{2}$	−0.041	−0.044	−0.046
	(−1.22)	(−1.22)	(−1.27)
${\;}_{△}Cash$	−0.867 ***	−1.111 ***	−1.154 ***
	(−3.74)	(−4.44)	(−4.64)
$U_{1}\times_{△}Cash$	3.752 ***		
	(4.40)		
$U_{3}\times_{△}Cash$		2.297 ***	
		(2.91)	
$U_{12}\times_{△}Cash$			1.432
			(1.52)
$U_{1}$	−0.461 ***		
	(−4.40)		
$U_{3}$		−0.282 ***	
		(−2.91)	
$U_{12}$			−0.176
			(−1.52)
LEV	−0.251	−0.319	−0.297
	(−1.07)	(−1.39)	(−1.29)
ROA	0.331	0.078	−0.043
	(0.60)	(0.13)	(−0.08)
Growth	0.135 ***	0.158 ***	0.159 ***
	(3.43)	(3.84)	(3.77)
TOP1	0.001	−0.003	−0.002
	(0.17)	(−0.39)	(−0.36)
Size	−0.324	0.062	0.116
	(−1.42)	(0.25)	(0.48)
Constant	4.387 *	0.764	−1.069
	(1.91)	(0.34)	(−0.49)
observations	774	774	774
${Adj\;R}^{2}$	0.220	0.197	0.187

Note: ***, * indicate significance levels of 1%, and 10%, respectively.

**Table 5:** Threshold estimates and confidence intervals.

	Threshold Estimates	95% Confidence Intervals
A single threshold	−13.7693	(−40.0616, −12.6846)
A double threshold	Not obviously	

Note: Confidence interval in () indicates the threshold is at a 95% confidence level.

**Table 6:** Threshold affect test results.

Variables	Model	F	P	BS	1%	5%	10%
RD	Single threshold	8.85	0.0790	1000	15.5161	10.7775	8.2041

**Table 7:** Financing constraints threshold model estimates.

Variables	Equation (5)
	RD
${RD}_{t-1}$	0.774 ***
	(8.84)
${RD}_{t}^{2}$	−0.048 ***
	(−4.41)
${\;}_{△}CashI(ICR\leq -13.7693)$	−2.224 ***
	(−4.61)
${\;}_{△}CashI(ICR\;\text{>}-13.7693)$	−0.679 **
	(−2.08)
LEV	−0.322
	(−1.18)
ROA	−0.142
	(−0.27)
Growth	0.150 ***
	(3.58)
TOP1	−0.002
	(−0.26)
Size	0.108
	(0.72)
Constant	−0.013
	(−0.01)
observations	774
${Adj\;R}^{2}$	0.194

Note: ***, ** indicate significance levels of 1% and 5%, respectively.

**Table 8:** The impact of monetary policy uncertainty on R&D investment smoothing behavior under different financing constraints.

Variables	Equation (4)					
	ICR≤−13.7693			ICR>−13.7693		
	RD			RD		
${RD}_{t-1}$	0.484	0.637	0.590	0.704 ***	0.731 ***	0.759 ***
	(1.26)	(1.39)	(1.31)	(5.64)	(5.88)	(5.95)
${RD}_{t}^{2}$	−0.034	−0.046	−0.041	−0.035	−0.036 *	−0.039 *
	(−0.57)	(−0.67)	(−0.61)	(−1.60)	(−1.72)	(−1.71)
${\;}_{△}Cash$	−1.394 **	−1.827 ***	−1.775 ***	−0.339	−0.599 **	−0.634 **
	(−2.56)	(−3.01)	(−2.94)	(−1.16)	(−2.00)	(−2.15)
$U_{1}\times_{△}Cash$	6.741 **			3.139 ***		
	(2.60)			(3.88)		
$U_{3}\times_{△}Cash$		5.038 *			2.043 **	
		(1.93)			(2.10)	
$U_{12}\times_{△}Cash$			4.410			0.809
			(1.21)			(0.82)
$U_{1}$	−0.828 **			−0.386 ***		
	(−2.61)			(−3.88)		
$U_{3}$		−0.619 *			−0.251 **	
		(−1.93)			(−2.10)	
$U_{12}$			−0.541			−0.099
			(−1.21)			(−0.82)
LEV	−0.054	−0.584	−0.626	−0.227	−0.275	−0.251
	(−0.04)	(−0.48)	(−0.50)	(−0.76)	(−0.94)	(−0.85)
ROA	−0.387	−1.579	−1.926	0.861	0.899	0.698
	(−0.23)	(−0.82)	(−1.02)	(1.28)	(1.36)	(1.08)
Growth	0.120	0.314	0.212	0.119 **	0.133 **	0.130 **
	(0.44)	(1.13)	(0.78)	(2.44)	(2.55)	(2.56)
TOP1	−0.020	−0.028	−0.026	0.004	−0.000	0.000
	(−0.61)	(−0.79)	(−0.72)	(0.65)	(−0.01)	(0.06)
Size	−0.539	0.076	0.380	−0.419	−0.025	0.004
	(−0.85)	(0.11)	(0.52)	(−1.56)	(−0.09)	(0.01)
Constant	8.072	2.104	−1.634	5.039 *	1.589	0.384
	(1.23)	(0.33)	(−0.23)	(1.87)	(0.59)	(0.16)
observations	199	199	199	575	575	575
${Adj\;R}^{2}$	0.135	0.108	0.088	0.293	0.273	0.261

Note: ***, **, * indicate significance levels of 1%, 5%, and 10%, respectively.

## Data Availability

Publicly available datasets were analyzed in this study. This data can be found here: [http://cn.gtadata.com, https://cas.ceicdata.com and https://db.cei.cn, all accessed date on 15 October 2020].

## Appendix A

**Table A1:** Explanation of variables used in constructing China’s monetary policy uncertainty index.

Name	Variable	Calculation Method and Description	Abbreviation
Monetary policy	Money supply M0	Year-on-year growth ratio	M0
	Money supply M1	Year-on-year growth ratio	M1
	Money supply M2	Year-on-year growth ratio	M2
	Required reserve ratio	Level value	DRR
	Rate of rediscount	Level value	DIR
	Lending rate: Within 1 year(including 1 year)	Level value	LR1
	Lending rate: 1–5 years	Level value	LR1–5
	Lending rate: More than 5 years	Level value	LR5
	Benchmark one-year deposit rate	Level value	DR1
	Benchmark two-year deposit rate	Level value	DR2
	Benchmark three-year deposit rate	Level value	DR3
	CHIBOR (China inter-bank offered rate): 7-day weighted average	Level value	ILR7
	CHIBOR (China inter-bank offered rate): 1-month weighted average	Level value	ILR1
	CHIBOR (China inter-bank offered rate): 3-month weighted average	Level value	ILR3
Bond market	1-year treasury bond term spread	The difference between the 1-year treasury bond term spread and 3-month treasury bond term spread	1y3mTS
	3-year treasury bond term spread	The difference between the 3-year treasury bond term spread and 3-month treasury bond term spread	3y3mTS
	5-year treasury bond term spread	The difference between the 5-year treasury bond term spread and 3-month treasury bond term spread	5y3mTS
	10-year treasury bond term spread	The difference between the 10-year treasury bond term spread and 3-month treasury bond term spread	10y3mTS
	1-year 3A enterprise bond term spread	The difference between the 1-year 3A enterprise bond term spread and 3-month treasury bond term spread	1y3mCS
	3-year 3A enterprise bond term spread	The difference between the 3-year 3A enterprise bond term spread and 3-month treasury bond term spread	3y3mCS
	5-year 3A enterprise bond term spread	The difference between the 5-year 3A enterprise bond term spread and 3-month treasury bond term spread	5y3mCS
	10-year 3A enterprise bond term spread	The difference between the 10-year 3A enterprise bond term spread and 3-month treasury bond term spread	10y3mCS
Securities market	The return ratio of Shanghai stock exchange composite index	Use the monthly Shanghai Composite Index to get the rate of return	R_SH
	The return ratio of Shenzhen stock exchange composite index	Use the monthly Shenzhen Composite Index to get the rate of return	R_SZ
	The fluctuation ratio of Shanghai stock exchange composite index	Get the conditional standard deviation of the stock exchange composite index yield from GARCH (1,1)	SVOL_SH
	The fluctuation ratio of Shenzhen stock exchange composite index	Get the conditional standard deviation of the stock exchange composite index yield from GARCH (1,1)	SVOL_SZ
	The turnover ratio of Shanghai stock exchange composite index	Average the daily turnover rate to get the monthly turnover rate	TO_SH
	The turnover ratio of Shenzhen stock exchange composite index	Average the daily turnover rate to get the monthly turnover rate	TO_SZ
Macroeconomics	Value-added of large industrial enterprises	Actual year-on-year growth rate	VAI
	Macro-economic climate index: leading index	Year-on-year growth ratio	MI1
	Macro-economic climate index: consistent index	Year-on-year growth ratio	MI2
	Macro-economic climate index: lagging index	Year-on-year growth ratio	MI3
	Macro-economic climate index: warning index	Year-on-year growth ratio	MI4
	Purchase management index: manufacturing	Year-on-year growth ratio	PMI
	Total retail sales of consumer goods	Year-on-year growth ratio	SCR
	Fixed asset investment: cumulative	Year-on-year growth ratio	FI
Price level	Consumer price index	Year-on-year growth ratio	CPI
	Retail price index of commodities	Year-on-year growth ratio	RPI
	Enterprise goods price index	Year-on-year growth ratio	FPI
	The producer price index	Year-on-year growth ratio	IPI
	Price index of agricultural means of production	Year-on-year growth ratio	API
Exchange rate market	RMB against US dollar	Level value	PBC
	Real effective exchange rate: BIS	Year-on-year growth ratio	BIS
Government spending	National general public budget revenue	Year-on-year growth ratio	GI
	National general public budget expenditure	Year-on-year growth ratio	GS
